# Excessive Lipid Production Shapes Glioma Tumor Microenvironment

**DOI:** 10.21203/rs.3.rs-3694185/v1

**Published:** 2023-12-12

**Authors:** Haitham Maraqah, John Paul Aboubechara, Mones Abu-Asab, Han Sung Lee, Orwa Aboud

**Affiliations:** An-Najah National University; University of California, Davis; National Eye Institute; University of California, Davis; University of California, Davis

**Keywords:** glioblastoma, astrocytoma, lipid accumulation, mitochondrial dysfunction, tumor microenvironment, oxidative phosphorylation, glycolysis, immune evasion, therapeutic strategies, ultrastructural analysis

## Abstract

Disrupted lipid metabolism is a characteristic of gliomas. This study utilizes an ultrastructural approach to characterize the prevalence and distribution of lipids within gliomas. This study made use of tissue from IDH1 wild type (IDH1-wt) glioblastoma (n = 18) and IDH1 mutant (IDH1-mt) astrocytoma (n = 12) tumors. We uncover a prevalent and intriguing surplus of lipids. The bulk of the lipids manifested as sizable cytoplasmic inclusions and extracellular deposits in the tumor microenvironment (TME); in some tumors the lipids were stored in the classical membraneless spheroidal lipid droplets (LDs). Frequently, lipids accumulated inside mitochondria, suggesting possible dysfunction of the beta-oxidation pathway. Additionally, the tumor vasculature have lipid deposits in their lumen and vessel walls; this lipid could have shifted in from the tumor microenvironment or have been produced by the vessel-invading tumor cells. Lipid excess in gliomas stems from disrupted beta-oxidation and dysfunctional oxidative phosphorylation pathways. The implications of this lipid-driven environment include structural support for the tumor cells and protection against immune responses, non-lipophilic drugs, and free radicals.

## 1. Introduction

Despite being the most common malignant brain tumors in adults, IDH1-wildtype glioblastoma and IDH1-mutant astrocytoma are not sufficiently studied ultrastructurally. We have recently shown that these tumors are ultrastructurally unique in their mitochondrial dysfunction and pattern of vessel invasion by tumor cells [1, 2]. As the mitochondria is important for beta-oxidation in lipid catabolism, we hypothesized that mitochondrial dysfunction in glioma cells would result in lipid accumulation. We report here using transmission electron microscopy (TEM) that glioma cells produce an excessive amount of lipids that are found broadly in many cellular structures and tumor microenvironment, including the cytoplasm, mitochondrial matrix, and nucleus of tumor cells, as well as spread to tumor vessel walls and lumina. Furthermore, we examine lysosome prevalence, glycogen droplets, plasma membrane integrity, and chromatin structure. We conclude with a discussion of the implications of our findings in the survival of tumor cells, as well as commenting on diagnostic and therapeutic implications of lipid accumulation.

**Figure 1:**
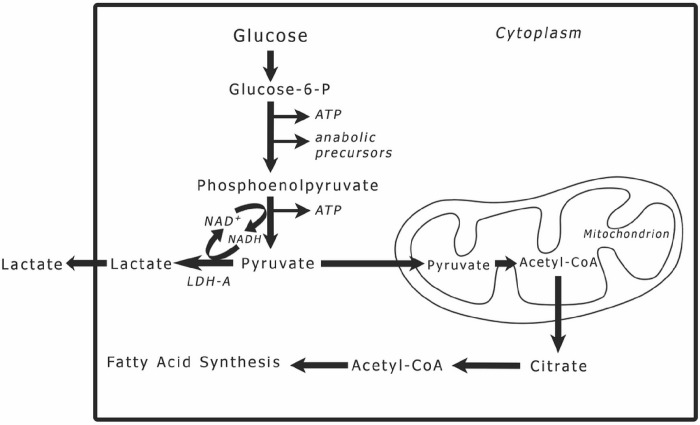
The fate of pyruvate in cancer cells. The end product of glycolysis is pyruvate, which can enter into two pathways. The first is to be reduced to lactic acid through an exothermic reaction catalyzed by lactate dehydrogenase-A (LDH-A); the second is to be converted into acetyl-CoA in the mitochondria and processed through the TCA cycle and oxidative phosphorylation. However, when oxidative phosphorylation is dysfunctional in a cancer cell and acetyl-CoA accumulates in the mitochondria, acetyl-CoA is exported to the cytoplasm and gets assimilated into fatty acid synthesis, the building blocks of lipids.

Metabolic reprogramming is a hallmark of cancer, and specifically excessive lipid production and accumulation is a prominent feature that has been reported in many cancers [3]. Lipids in cancer is a subject that has been attracting increasing attention in cancer pathogenesis. Lipid content in the cell or tissue is linked to glucose metabolism. Pyruvate, the end product of glycolysis (Fig. 1), is directly responsible for fatty acid production. In a tumor cell, pyruvate may either be reduced to lactic acid in the cytoplasm or converted to acetyl-CoA in the mitochondria [4]. When the process of oxidative phosphorylation in the mitochondria is dysfunctional, the acetyl-CoA is exported outside the mitochondria and used for fatty acid (FA) synthesis [5]. To avoid lipotoxicity, tumor cells convert FAs into diacylglyceride and triacylglyceride lipids via the glycerol phosphate pathway, which uses the glycolytic intermediate glycerol-3-phosphate to form the glycerol backbone of these lipids by increasing the expression of diacylglycerol acyltransferase 1 (DGAT1) [6, 7]. Additionally, acetyl-CoA synthetase 2 (ACSS2) converts cytosolic acetate to acetyl-CoA for de novo FA and cholesterol synthesis [7].

FAs are an energy substrate of normal cells, and only under hypoxic or other forms of stressful conditions does lactate production become a more significant source of ATP [8]. On the other hand, primary tumors are known to produce lactic acid from pyruvate (the Warburg Effect). Cancer cells shift their preference to FA production and generate excessive amounts of FAs, cholesterol, and lipids in general, and this is also the case in gliomas. The irony in such a switch is that these types of tumors are known to have uncoupled (depolarized) mitochondrial membranes and a dysfunctional oxidative phosphorylation, and thus, cannot utilize FAs for ATP production [1]. This prompts the question of what is the significance of producing insoluble FAs over the soluble lactic acid? Do FAs give a selective advantage to tumor cells, or is it a metabolic short circuit that does not confer any fringe benefit on the cancer cells?

In this study, we performed a comprehensive ultrastructural analysis of lipid accumulation inside tumor cells and in the tumor microenvironment of two types of gliomas: IDH1-mt astrocytoma and IDH1-wt glioblastoma. The amount of lipids and their cellular distribution pattern constitute a unique phenomenon for these tumors; this aspect of carcinogenesis has not been well characterized ultrastructurally by previous studies. By using electron microscopy, we revealed the physical features and biochemical phenotype of these lipidized gliomas. Some aspects of this work could explain the tumors’ aggressiveness and resistance to treatment. In addition to providing new insights into the pathophysiology of this devastating disease, our study may offer new ideas for diagnosis and treatment strategies.

## 2. Materials and Methods

### Tissue Specimens

All 30 brain tumor tissue specimens were obtained from patients with histopathological confirmed high-grade glioma. The study protocol was approved by the Institutional Review Board of The University of California Davis. The study utilized specimens of formalin-fixed paraffin-embedded tumors. The specimens represented 12 tumors of IDH1-mt astrocytoma and 18 tumors of IDH1-wt glioblastoma. The tissues were dug out of the paraffin blocks after examining their hematoxylin and eosin (H&E) slides and determining the areas of the tumor viable cells.

### Tissue Preparation, Image Acquisition, and Descriptive Analysis

The specimens were deparaffinized in xylene overnight followed by one more change of xylene for 20 min, two changes of absolute ethanol (5 min each), one change of 70% ethanol (5 min), and three changes of phosphate buffered saline (PBS, 5 min each), then processed for transmission electron microscopy (TEM) according to Abu-Asab [9]. Briefly, specimens were washed in PBS, post-fixed in 0.5% osmium tetroxide (OsO_4_), rinsed, dehydrated, then embedded in Spurr’s epoxy resin. Blocks were sectioned at ~ 90 nm thickness on a Leica EM UC6 ultramicrotome (Leica, Austria), double-stained with uranyl acetate and lead citrate, and imaged with JEOL JEM-1010 electron microscope (JEOL, Japan). Surveys of the tissues were made with descriptive analysis done for the observed ultrastructure.

## 3. Results

### 3.1. The State of Lipids in Glioma Tumors

**Figure 2:**
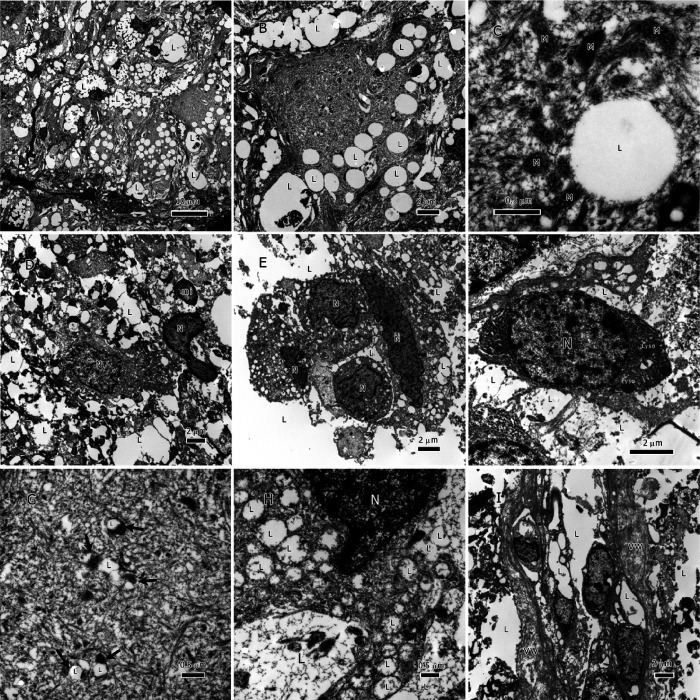
Lipids in IDH1-WT glioblastoma tumors. A-C, lipid droplets inside tumor cells, lipid droplets fuse with each other and form larger droplets; C. a single lipid droplet surrounded by degenerate mitochondria, the cytoplasm is full of lipid and intermediate filaments. D-F, an overview of lipids interspersed between tumor cells in addition to lipid droplets inside tumor cells. G & H, lipid inside mitochondria that are in different degrees of degeneration, arrows in G point to the degenerate mitochondria. I, lipids within the vessel lumen, all cells in this image are tumor cells invading the vessel. Abbreviations: Lyso=Lysosome, L=lipid, M=mitochondrion, mi=myelin inclusion, N=nucleus, VW=vessel wall.

**Figure 3:**
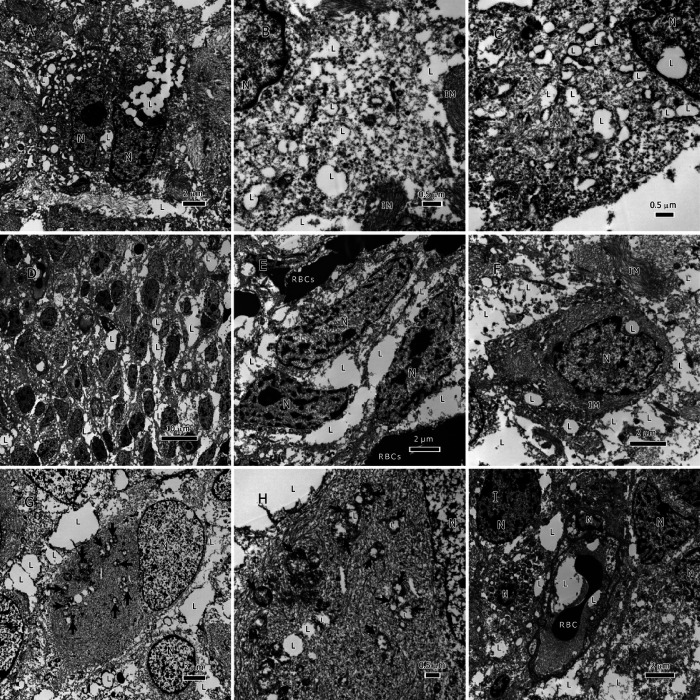
Lipids in IDH1-mt astrocytoma tumors. A-C, lipid droplets (LDs) inside tumor cells, B, LDs at a higher magnification inside the cytoplasm, C, LDs that were formed by filling up the degenerate mitochondria, notice the remaining mitochondrial membrane, a LD present inside the nucleus. D-F, an overview of lipids interspersed between tumor cells in addition to LDs inside tumor cells. G & H, lipid inside mitochondria that are in different degrees of degeneration, arrows point to the degenerate mitochondria, the cytoplasm is packed with intermediate filaments. I, lipids within the vessel lumen. Abbreviations: IM=intermediate filaments, L=lipid, N=nucleus, RBC=red blood cell.

**Figure 4:**
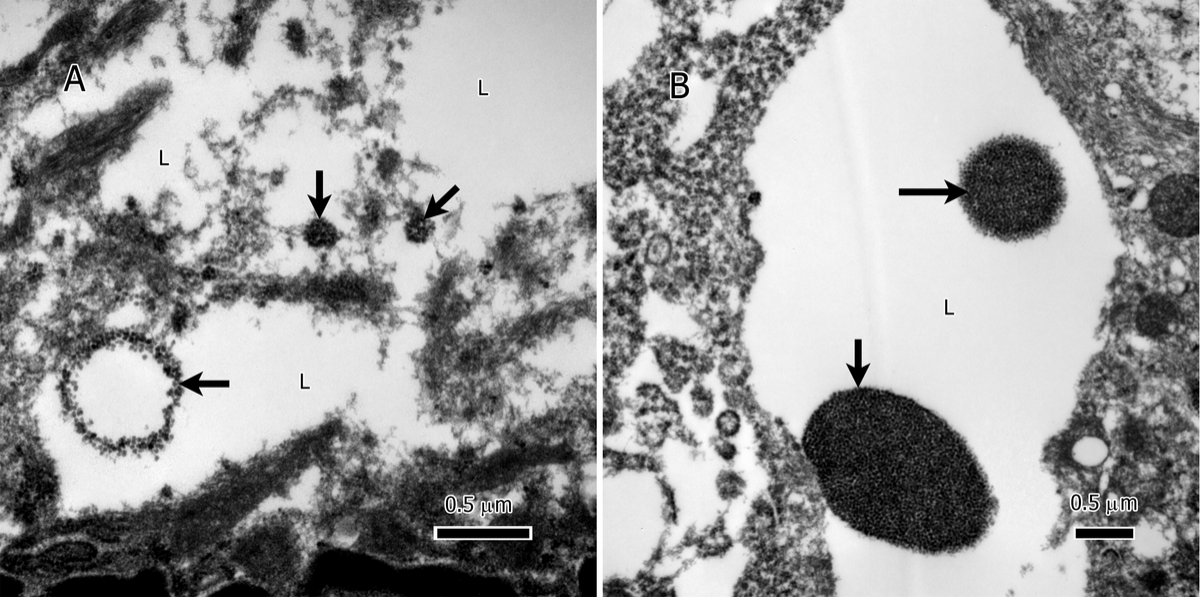
Extranuclear DNA (enDNA) fragments (black arrows) embedded in the lipid deposits of IDH1-mt astrocytoma tumor cells. A, a large chromatin circle (~750 nm in diameter) and two small chromatin clumps (the smallest 125 nm in the largest dimension). B, two large chromatin clumps in the lipid deposits, the large clump is ~1.75 μm, and the small one ~750 nm. Abbreviations: L=lipid.

Lipids in glioma tumors exist as either the classical membraneless spheroidal lipid droplets (LDs) or as large amorphous intracellular inclusions or extracellular deposits (Figs. 2, 3 & 4). LDs were found in both glioma types; however, IDH1-wt glioblastoma had more classical LDs in its tumor cells than the IDH1-mt astrocytoma tumors. Regularly, LDs seemed to have fused with each other and made large bodies of intracellular lipid inclusions (Figs. 2A & 2B, 3A & 3B). In addition to LDs, most of the tumor lipids were in the form of cytoplasmic amorphous inclusions, intramitochondrial lipids (Figs. 2G & 2H, and 3G & 3H), intravascular in the tumor neovascularization (Figs. 2I & 3I), and extracellular deposits between tumor cells (i.e., in the tumor microenvironment; Figs. 2D–F, and 3C–F). The nuclei seemed to be lipidized and occasionally contain LDs (Fig. 3C).

Lipid accumulation inside mitochondria was found in both glioma types (Figs. 2G & 2H, and 3G & 3H). Because damaged mitochondria without lipids were previously reported in these glioma tumors [1], it is unlikely that the lipid accumulation inside the mitochondria has caused the destruction of the inner membrane and the subsequent formation of a membrane-bound lipid droplets. The mitochondria-derived lipid droplets can be distinguished from the classical LDs (see Fig. 2C for a classical LD) by the presence of the outer mitochondrial membrane around them. No membrane-bound LDs were found in the tumor microenvironment. There were many single-membraned mitochondrial vesicles in the cytoplasm that resulted from the destruction of the inner membranes of the mitochondria, these give the appearance of autophagosomes (Fig. 2H and 3C) and considered as such.

### 3.2. Lysosomes and Glycogen

On characterization of lysosome prevalence in both glioma types, we observed that these organelles were occasionally seen but were not ubiquitous in the tumors (Fig. 2F). We also surveyed for glycogen granules, and found scant amounts of these structures in some tumors’ cells.

### 3.3. The State of the Plasma Membrane of Tumor Cells

In both glioma types, tumor cells embedded in lipids appeared to be lacking their plasma membranes; this is a phenomenon that was also observed in metastatic breast cancer cells surrounded by excessive amounts of lipids.[10] Thus far, it is unclear if the lipids have caused the destruction and disappearance of the plasma membranes or if the tumor cells use the new lipid shield surrounding them as a functional membrane. Loss of cellular membranes could be a feature of the metastatic phenotype.

### 3.4. Chromatin Fragmentation in Astrocytoma

Intriguingly, within the lipid deposits of IDH1 mutant (IDH1-mt) astrocytoma tumor cells, our study identified extranuclear DNA (enDNA) fragments (Fig. 4A & B). These enDNA fragments, originating from nuclear chromatin, were observed in various sizes and exhibited two distinct shapes: circular (Fig. 4A, lower left) and clumped structures (Fig. 4A & B). This phenomenon, also referred to as cytoplasmic chromatin fragments (CCF) or abnormal chromatin clumps, highlights an additional layer of complexity in the lipid-rich microenvironment of these tumors.

## 4. Discussion

Metabolic reprogramming of lipogenesis is a salient feature of tumor cells. One aspect of this reprogramming is evident in many cancers as lipid overproduction and abnormal distribution. Lipid accumulation and storage take several forms that include lipid droplets (membraneless and membranous), cytoplasmic amorphous inclusions, intramitochondrial lipids, nuclear deposits, extracellular deposits between tumor cells (i.e., in the tumor microenvironment), and intravascular—particularly in tumor neovascularization. This phenomenon of lipid overproduction has been shown in glioma tumors and breast cancer[11]. As described below, lipid accumulation has myriad effects on cancer cell metabolism and function, while also posing important obstacles for treatment of patients with gliomas.

### Causes of Lipid Accumulation in Tumor Cells

Three mechanisms have been elucidated that can promote lipid accumulation in tumor cells. First, oxidative phosphorylation is often compromised in the mitochondria of tumor cells, which results in a net flux of metabolites towards acetyl-CoA production and subsequent fatty acid synthesis [12]. These lipids can then behave as uncouplers and inhibitors of oxidative phosphorylation [12, 13], further driving excess fatty acid synthesis. Second, dysfunctional oxidative phosphorylation forces tumor cells to rely on glycolysis for ATP production. This hyperactive glycolysis results in the accumulation of negatively charged lactic acid, which reduces the release of fatty acids from tumor cells [14]. Third, as described in the next section below, lipid accumulation can result in a hypoxic tumor microenvironment, which further impairs oxidative phosphorylation and resultant increased flux of metabolites into lipid production.

### Defective Fatty Acid Oxidation Protects Against Oxidative Damage

Fatty acid oxidation can lead to the development of reactive oxygen species (ROS), which are generally toxic to cells [12]. Tumor cells have impaired fatty acid oxidation, and this results in a reduction in ROS formation in tumor cells, thereby protecting the cells from oxidative stress [12]. As a result, lipid accumulation serves a pro-tumorigenic function by further inhibiting fatty acid oxidation and decreasing toxic ROS concentrations.

### Excessive Tumor Lipids Enforce a Hypoxic Microenvironment

Lipid accumulation in glioma tumors can insulate and hence reduce the concentration of oxygen that reaches cancer cell mitochondria, resulting in a hypoxic tumor microenvironment. Furthermore, cholesterol—a component of lipidized tumors—is known to act as a negative modulator of oxygen diffusion into cells [15]. Although the amount of cholesterol in cancer cells may vary depending on the type of cancer and its stage, cancer cells have about twice as much cholesterol as normal cells[16], and this could be even higher in gliomas where the tumor cells have sizable intracellular lipid storage and are embedded in a lipid-overloaded microenvironment[17]. As a result, lipid and specifically cholesterol accumulation in tumor cells may partially explain the dysfunctional and degenerate state of their mitochondria.

### Lipids Role in Cancer Immune Evasion

Cancer cells are capable of secreting lipid metabolites that alter the functions of immune cells, and thus, creating an immune-challenging tumor microenvironment [18, 19]. As a matter of fact, the tumor microenvironment of the examined gliomas was free of immune cells. Multiple well-pronounced immunosuppressive mechanisms have been reported in glioblastoma that could pose significant challenges to the application of immunotherapy for glioma treatment. First, gliomas express FAS-Ligand that could induce FAS-L/FAS-mediated apoptosis of lymphocytes in the brain [20]. Second, T-lymphocyte checkpoints can be triggered through PD-L1 surface expression in glioblastoma. Third, glioma cells have permanent expression of the immunosuppressive molecules TGFβ1 and TGFβ2 [21]. These immunosuppressive mechanisms can enable glioblastoma cells to evade immune detection and cause the destruction of immune cells, thus allowing for unchecked growth and proliferation within the brain. Furthermore, recent studies have indicated that glioblastoma cells produce lipids and FAs that act as signaling molecules that enable cancer cells to either evade detection by the immune system or actively suppress the immune response [18]. Consequently, despite advancements in tumor immunotherapy, the aforementioned defensive mechanisms suggest that the ongoing immunotherapy trials may not be sufficiently effective and other therapeutic strategies will be needed to better target glioblastoma cells.

### Lipids and Autophagy

Despite reports of autophagosome involvement in FAs and lipid extracellular trafficking [22, 23], no autophagosomes were observed in both cancer types in our survey. However, there were many mitochondrial vesicles in the cytoplasm that resulted from the destruction of the inner membranes of the mitochondria; some of these were filled with lipids (Fig. 2H and 3C). Single membrane-bound mitochondrial vesicles, whether carrying lipids or not, are homologous to autophagosomes [24], and depending on the detection method used, they could give the same signal as autophagosomes.

### Effect of Lipids on Tumor Temperature

Tumors tend to have different temperatures than their normal counterparts. While lactic acid production tends to increase the tumor temperature because it is an exothermic reaction that produces 25.1 kJ/mol (ΔG’°=−25.1 kJ/mol), lipid production does not, and it has the opposite effect [25]. Therefore, tumors producing excessive amounts of FAs in their microenvironment are expected to be cooler. For example, lipomatous glioma brain tumors tend to be hypothermic or lower in temperature than the surrounding healthy tissues [26]. Some reports suggested treating glioma tumors with hypothermia [27]; however, an opposite trend is making headway to treat malignant glioma using hyperthermia [28]. We think that hyperthermia is probably more appropriate since it may strip the tumor and its cells from their protective fatty coat.

### Diagnostic Implications of Tumor Lipids

Tumor lipids have the potential to serve as valuable diagnostic tools due to their ability to migrate into tumor vessels (Figs. 2I and 3I) and eventually enter the general circulation. By comparing serum lipidomics with established baselines, it could be feasible to monitor tumor recurrence. To accomplish this, it is crucial to create reference points of lipids for healthy individuals, patients with primary tumors, and patients experiencing recurrence. Furthermore, investigating the possibility of using specific lipids that enter the serum as biomarkers for monitoring tumor recurrence is worthy of further exploration.

### Inflammatory Consequences of Extranuclear DNA

Cancer cells are known to harbor extranuclear DNA (enDNA) in their cytoplasm [29]. Extranuclear DNA has been shown to play an important role in tumors through increased oncogene expression, genetic heterogeneity between cancer cells, and resistance to therapy [30].The presence of double-stranded DNA (dsDNA) in the cytoplasm (Fig. 4) triggers the cGAS-STING signaling pathway. When detecting dsDNA, the cGAS produces cGAMP (cyclic GMP-AMP) which acts through STING to induce an inflammatory response in the form of a cytokine cascade and type I interferons (IFNs) [31]. However, when the enDNA is embedded in lipids as is the case in the examined gliomas (Fig. 4), the enDNA may be hidden from cGAS and would not elicit the same inflammatory response.

### Therapeutic challenges of lipid accumulation

Glioblastoma lipid accumulation poses important consequences for tumor growth and therapeutic resistance [7]. Lipid accumulation in tumor cells presents an interesting therapeutic challenge. Lactic acid is produced from pyruvate, the final product of the glycolytic pathway; however, for an unknown reason there is a later metabolic shift towards producing acetyl-CoA from pyruvate instead of lactic acid [32]. This shift in the pyruvate fate could be considered a crucial point in cancer phenotypic transition from a predominant acid-producing phenotype to a neutral-lipidic phenotype. This change in metabolite state of tumor cells suggests that such a shift requires a change in the therapeutic strategy. Because the brain is a lipid-rich organ, the brain’s tumors are mostly treated with lipophilic drugs to ensure their reach to the tumor sites. Unfortunately, the lipid rich tumors will act as a sink sequestering hydrophobic therapeutic agents, and thus diminishing their effectiveness [33].

### New Therapeutic Strategies for Lipidized Tumors

The excessive lipid production and accumulation observed in these glioma tumors serves as both a limitation of current treatments, but also holds promise for the development of new therapeutics [6]. Specifically, there are four strategies to explore within the lipid-therapy paradigm. The first strategy is to disrupt tumor metabolism through altering tumor lipid production. This approach has been employed in breast cancer, wherein it was shown that inhibition of fatty acid synthesis inhibited cancer cell growth in brain metastases[11]. For gliomas, additional work is needed to understand the aforementioned extracellular lipid shield that protects tumor cells from immune cells. The second strategy is to explore whether the lipids produced by gliomas can be identified in the serum, and whether a detailed understanding of these circulating lipids could allow for the development of plasma biomarkers of tumor recurrence. The third strategy is the development of a more effective lipophilic drug delivery system. In lipidized tumors, the issue of effective drug penetration of the tumor is a serious challenge. However, with recent success of the liposome-based vaccines for immunization against SARS COVID-19 [34], the idea of a liposome drug delivery against lipidized tumors seems like a strategy worth exploring [35]. This approach will allow for increased effective concentrations of chemotherapy in tumor cells, and potentially allow for new classes of drugs to be effective for gliomas.

The fourth strategy is to reduce the immune suppressive effect of the lipids on immune cells. Lipids produced by glioma tumors can act as signaling molecules, allowing the cancer cells to evade detection by the immune system or actively suppress it [36]. Therefore, tumor-mediated immunosuppression presents a significant challenge in developing effective therapies for these types of tumors. This will also be important as immunotherapy with novel chimeric antigen receptor (CAR) T cells have shown promise in eliminating human glioblastoma cells transplanted into the brains of mice [37]. Studies will be needed to explore how these immunosuppressive lipids interact with the CAR T cells. A better understanding of the role of lipids in tumor-mediated immunosuppression is crucial to developing effective novel therapies that can improve prognosis.

## 5. Conclusions

The ultrastructural analysis presented in this study provides valuable insights into lipid accumulation and distribution within IDH1-wt glioblastoma and IDH1-mt astrocytoma tumors as well as their implications on tumor survival and aggressiveness. We demonstrate that gliomas have a lipidized phenotype with lipid accumulation in intracellular lipid droplets, within mitochondria, extracellular lipid deposits, and even intravascularly. A deeper understanding of the mechanisms driving lipidized glioma tumor formation will enable an understanding of tumor pathogenesis, treatment resistance and cancer immune evasion. Further investigation is also needed to probe if plasma lipids can be used as early biomarkers for detection of cancer recurrence and prognosis. As current treatments are of limited efficacy, this novel understanding of lipids in gliomas must be exploited to develop new treatments to improve patient outcomes.

## Data Availability

Data can be requested by contacting the corresponding author.
